# Ethnic differences in insulin sensitivity and beta-cell function among Asian men

**DOI:** 10.1038/nutd.2015.24

**Published:** 2015-07-20

**Authors:** V M H Tan, Y S Lee, K Venkataraman, E Y H Khoo, E S Tai, Y S Chong, P Gluckman, M K S Leow, C M Khoo

**Affiliations:** 1 Singapore Institute for Clinical Sciences, Agency for Science, Technology and Research (A*STAR), Singapore; 2 Department of Paediatric, Yong Loo Lin School of Medicine, National University of Singapore, Singapore; 3 Division of Paediatric Endocrinology and Diabetes, Khoo Teck Puat-National University Children's Medical Institute, National University Hospital, National University Health System, Singapore; 4 Saw Swee Hock School of Public Health, National University of Singapore, Singapore; 5 Department of Medicine, Yong Loo Lin School of Medicine, National University of Singapore, Singapore; 6 Division of Endocrinology, Department of Medicine, National University Health System, Singapore; 7 Duke-NUS Graduate Medical School, Singapore; 8 Obstetrics and Gynaecology, Yong Loo Lin School of Medicine, National University of Singapore, Singapore; 9 Liggins Institute, Auckland, New Zealand; 10 Department of Endocrinology, Tan Tock Seng Hospital, Singapore

## Abstract

**Background and objectives:**

Lean Asian Indians are less insulin sensitive compared with Chinese and Malays, but the pancreatic beta-cell function among these ethnic groups has yet to be studied in depth. We aimed to study beta-cell function in relation to insulin sensitivity among individuals of Chinese, Malay and Asian-Indian ethnicity living in Singapore.

**Subjects and methods:**

This is a sub-group analysis of 59 normoglycemic lean (body mass index (BMI) <23 kg m^−^^2^) adult males (14 Chinese, 21 Malays and 24 Asian Indians) from the Singapore Adults Metabolism Study. Insulin sensitivity was determined using fasting state indices (homeostatic model assessment—insulin resistance), the euglycemic-hyperinsulinemic clamp (ISI-clamp) and a liquid mixed-meal tolerance test (LMMTT) (Matsuda insulin sensitivity index (ISI-Mat)). Beta-cell function was assessed using fasting state indices (homeostatic model assessment—beta-cell function) and from the LMMTT (insulinogenic index and insulin secretion index). The oral disposition index (DI), a measure of beta-cell function relative to insulin sensitivity during the LMMTT, was calculated as a product of ISI-Mat and insulin secretion index.

**Results:**

Asian Indians had higher waist circumference and percent body fat than Chinese and Malays despite similar BMI. Overall, Asian Indians were the least insulin sensitive whereas the Chinese were most insulin sensitive. Asian Indians had higher beta-cell function compared with Chinese or Malays but these were not statistically different. Malays had the highest incremental area under the curve for glucose during LMMTT compared with Asian Indians and Chinese. However, there were no significant ethnic differences in the incremental insulin area under the curve. The oral DI was the lowest in Malays, followed by Asian Indians and Chinese.

**Conclusion:**

Among lean Asians, Chinese are the most insulin sensitive whereas Asian Indians are the least insulin sensitive. However, Malays demonstrate higher postprandial glucose excursion with lower beta-cell response compare with Chinese or Asian Indians. The paths leading to type 2 diabetes mellitus might differ between these Asian ethnic groups.

## Introduction

Type 2 diabetes mellitus (T2DM) is projected to affect nearly 600 million people worldwide by 2035,^1^ with China and India projected to contribute more cases of T2DM than any other country in the world. The population of Singapore comprises three major ethnic groups; Chinese, Malays and Asian Indians. The prevalence of T2DM in Singapore has increased from 8.2% in 2004 to 11.3% based on the Singapore National Health Survey 2010.^2^ The highest prevalence of T2DM has been seen in Asian Indians (17.2%), followed by Malays (16.6%) and Chinese (9.7%).^2^

Much of the work to understand ethnic differences in susceptibility to T2DM has focused on insulin resistance.^3, 4, 5^ Recently, we showed that degree of insulin sensitivity differs between Asian ethnic groups and this ethnic difference is more prominent amongst lean individuals.^6^ Among lean and young Singaporean males, Chinese and Malays are more insulin sensitive compared with Asian Indians.^6, 7^

It is well established that the pathogenesis of T2DM involves both decreased insulin sensitivity and impaired beta-cell function.^8, 9^ Initially, decreased insulin sensitivity may be compensated by increased beta-cell response, and progression to T2DM is thought to occur when beta-cells fail to compensate to a sufficient degree.^10^ Accumulating evidence demonstrates that insulin secretory defect may play a more important role than insulin sensitivity in the pathogenesis of T2DM, particularly in East-Asians.^11, 12, 13^ However, studies comparing beta-cell function between Asian ethnic groups are limited. It is not clear whether Chinese, Malays and Asian Indians exhibit differences in beta-cell function, in addition to the differences in insulin sensitivity.

To gain further insights into the ethnic susceptibility to T2DM, the present study aimed to examine ethnic differences in beta-cell function among lean individuals of Chinese, Malay and Asian-Indian ethnicity, after accounting for differences in insulin sensitivity.

## Subjects and methods

### Subjects

This was a sub-group analysis of the Singapore Adults Metabolism Study (SAMS).^6^ SAMS was a cross-sectional study that examined the associations between ethnicity, obesity and insulin resistance in 3 ethnic groups involving 100 Chinese, 80 Malays and 78 Asian-Indian males. A more detailed account of the selection procedure and the study participants was elsewhere.^6^ The main study (SAMS) was registered at clinicaltrials.gov as NCT00988819.

Fifty-nine healthy lean (body mass index (BMI) <23 kg m^−^^2^) adult males, comprising 14 Chinese, 21 Malays and 24 Asian Indians, who had full data from the euglycemic-hyperinsulinemic clamp procedure and the liquid mixed-meal tolerance test (LMMTT) were included in this study. We selected lean individuals as we have shown previously that ethnic differences in insulin sensitivity was observed among lean but not overweight or obese individuals.^6^ All subjects had fasting blood glucose of <7.0 mmol l^−1^ and had no prior history of hypertension or dyslipidemia. We excluded those with significant changes in diet or weight loss of more than 5%, a history of heart disease, epilepsy, insulin allergy, current smoking, a history of ingesting any drug known to alter insulin sensitivity (for example, corticosteroids), or any hospitalization or surgery during the 6 months before enrollment in the study. Ethics approval was obtained from the National Healthcare Group Domain Specific Review Board (Singapore) (approval code number C/2009/00022). All subjects provided written informed consent.

### Clinical measurements

Demographic data, medical and drug history, and data on lifestyle factors were collected using interviewer-administered questionnaires. Height was measured using a wall-mounted stadiometer, and weight using a digital scale (SECA, model 803; Vogel & Halke, Hamburg, Germany). BMI was calculated using the weight (kg) divided by the square of height (m). Waist circumference was measured at the midpoint between the lower costal margin and iliac crest at mid-respiration. Body composition (percent body fat and lean body mass) was measured using a dual-energy X-ray absorptiometry scanner (Hologic Discovery Wi, Hologic, Bedford, MA, USA). All dual-energy X-ray absorptiometry measurements were performed within 1 week of the euglycemic-hyperinsulinemic clamp procedure and the LMMTT.

### Euglycemic-hyperinsulinemic clamp

Insulin sensitivity was assessed using the euglycemic-hyperinsulinemic clamp technique, after an overnight fast (10 h).^14^ Insulin was infused at a fixed rate of 40 mU m^−^^2^ body surface area/min for the duration of the clamp (120 min). Blood glucose was measured every 5 min using the glucose oxidase method (Yellow Spring Glucose Analyzer; YSI Life Sciences, Yellow Spring, OH, USA). The infusion rate of the dextrose 20% solution was adjusted to maintain a constant blood glucose level at about 90 mg dl^−1^ (5 mmol l^−1^) throughout the clamp. The insulin sensitivity index (ISI-clamp) was calculated using the mean glucose infusion rate and steady-state insulin concentrations during the final 30 min of the clamp, adjusted for the lean body mass.

### LMMTT procedure

On a separate day after the clamp procedure, the LMMTT was conducted after an overnight 10-h fast. As a mixed-nutrient load, the liquid mixed meal provides a more physiologic stimulus than glucose alone for assessing postprandial glucose and insulin responses,^15^ dynamically reflects beta-cell function^16^ and provides an appropriate stimulus for assessing insulin sensitivity.^17, 18^ Subjects were provided a liquid meal that consisted of two 200 ml servings of Ensure Plus (Abbott Laboratories, Columbus, OH, USA), each providing 300 kcal, 40.4 g carbohydrate, 9.8 g fat and 12.5 g protein. A single intravenous catheter was placed in the antecubital space for collection of venous blood. Blood samples were obtained from the indwelling catheter for plasma glucose and insulin concentrations at 0, 30, 60, 90, 120 and 240 min.

### Biochemical analyses

Biochemical analyses were conducted at the National University Hospital Referral Laboratory, which is accredited by the College of American Pathologists. Plasma glucose concentrations obtained during LMMTT were analyzed using enzymatic methods (ADVIA 2400, Bayer Diagnostics, Tarrytown, NY, USA), and plasma insulin concentrations using a chemiluminescence assay (ADVIA Centaur Analyzer, Siemens Healthcare Diagnostics, Tarrytown, NY, USA).

### Derivatives of insulin sensitivity and beta-cell function

Using fasting indices, we computed homeostatic model assessment—insulin resistance (HOMA-IR) using the formula: (fasting insulin (mU l^−1^) × fasting glucose (mmol l^−1^)/22.5). Homeostatic model assessment—beta-cell function (HOMA-B), a measure of beta-cell function was computed using the formula: (20 × fasting insulin (mU l^−1^))/(fasting glucose (mmol l^−1^)–3.5).

From the LMMTT, we calculated total area-under-the-curve (AUC) and incremental AUC (IAUC) for glucose and insulin responses using the trapezoidal rule.^19, 20^ The insulin secretion index, calculated as the ratio of total AUC insulin to total AUC glucose, provides information on the total insulin response following the LMMTT. The Matsuda insulin sensitivity index (ISI-Mat) was calculated as follows: 10 000/square root of (fasting glucose × fasting insulin) × (IAUC_glucose240_ × IAUC_insulin240_).^21, 22^ ISI-Mat is a measure of glucose disposal during the LMMTT, representing a composite of both hepatic and muscular tissue insulin sensitivity.^21^

The insulinogenic index was used as a marker of early-phase insulin response and was calculated as follows: (Insulin30–Insulin0)/(Glucose30–Glucose0). The oral disposition index (DI), a measure of beta-cell function relative to insulin sensitivity during the LMMTT, was calculated as a product of ISI-Mat and insulin secretion index.

### Statistical analyses

Data are presented as mean (SE) unless otherwise stated. Analysis of Variance was used for comparisons of continuous variables, with post hoc Bonferroni corrections applied for group comparisons. The incremental changes in the post-meal plasma glucose and insulin concentrations between ethnic groups were assessed using repeated-measures Analysis of Variance, with ethnic group set as between-subject factors and time as a within-subject factor. All analyses were carried out using the SPSS statistical analysis software version 19.0 (SPSS, Chicago, IL, USA) and adjusted for age. A *P*-value of <0.05 was considered statistically significant.

## Results

The mean (SE) for age and BMI was 25.9 (0.6) years and 21.3 (0.2) kg m^−^^2^, respectively. Table 1 shows the baseline characteristics of study participants by ethnic groups. Chinese were significantly older than Malays and Asian Indians, thus all subsequent analyses were age adjusted. The BMI was not statistically different between the ethnic groups, but Asian Indians had significantly higher waist circumference and percent body fat compared with Chinese or Malays. Fasting glucose concentrations were similar between the ethnic groups, but fasting insulin concentrations appeared higher among Asian Indians compared with Chinese and Malays (*P*>0.05). Other metabolic parameters (total cholesterol, LDL-cholesterol, HDL-cholesterol, triglycerides, AST and ALT) were not statistically different between ethnic groups.

### Glycemic and insulin response

**Figure 1:**
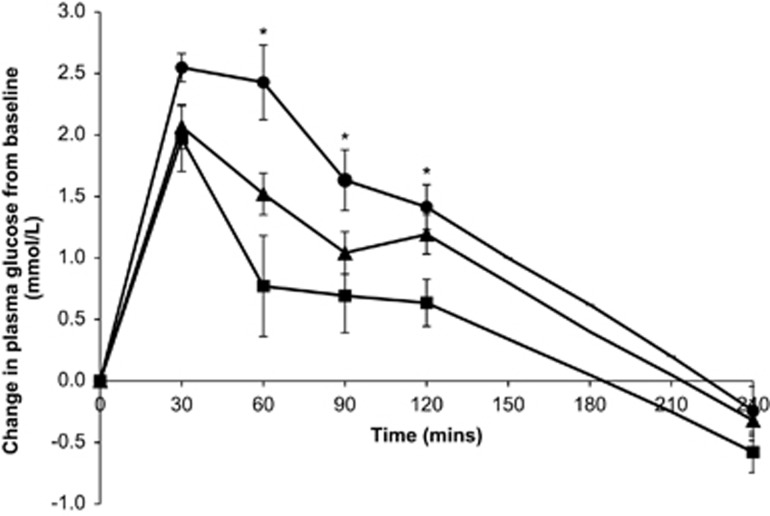
Incremental change in the plasma glucose concentration following LMMTT for Chinese (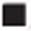), Malays (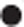) and Asian Indians (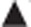). P interaction for ethnic groups × plasma glucose response=0.011 (adjusted for age). There were significant differences in the plasma glucose concentration at 60 min between Chinese and Malays (*P*<0.001); at 90 min between Chinese and Malays (*P*=0.010); at 120 min between Chinese and Malays (*P*=0.006), and at 120 min between Chinese and Asian Indians (*P*=0.039).

The overall total and IAUC for glucose were greatest in Malays, followed by Asian Indians and Chinese (*P*<0.05; Table 2). Figure 1 shows the incremental change in plasma glucose concentration following the LMMTT. The post-meal glycemic excursion displayed a time by ethnic group interaction (*P*_interaction_=0.011, adjusted for age).The plasma glucose concentrations were significantly higher in Malays compared with Chinese at 60 min and 90 min, and higher in Malays and Asian Indians compared with Chinese at 120 min.

There were no statistical differences in total or IAUC for insulin between the ethnic groups (Table 2), although Malays appeared to have lower insulin response compared with Chinese or Asian Indians. There was no significant difference in the post-meal plasma insulin responses between ethnic groups (*P*_interaction_=0.164; Supplementary Figure 1).

### Measures of insulin sensitivity

Asian Indians were the least insulin sensitive compared with Chinese or Malays, based on ISI-clamp and HOMA-IR (Table 2). The trend for ISI-Mat was similar to ISI-clamp, being highest in Chinese and lowest in Asian Indians. Similarly, adjusted ISI-clamp or ISI-Mat for waist circumference was lower in Asian Indians compared with Chinese. Insulin sensitivity was no different between Chinese and Malays based on the euglycemic clamp, but was significantly lower in Malays compared with Chinese based on the LMMTT derivative.

### Measures of pancreatic beta-cell function

In parallel with HOMA-IR, Asian Indians showed a significantly higher HOMA-B compared with Chinese and Malays (Table 2), indicating a compensatory hyperinsulinemia in the presence of greater insulin resistance to maintain fasting normoglycemia. Chinese had higher insulinogenic index (marker of early-phase insulin response) than Malays or Asian Indians, but Asian Indians had higher insulin secretion index (total insulin response following the LMMTT) than Chinese or Malays. The ethnic differences in insulinogenic index and insulin secretion index however, did not reach statistical significance.

### Relationship between insulin sensitivity and pancreatic beta-cell function

**Figure 2:**
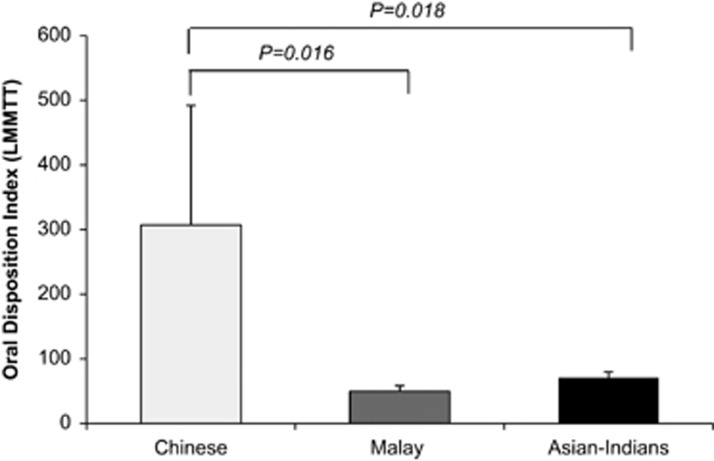
Mean (SE) oral DI derived from the LMMTT by ethnic group.

**Figure 3:**
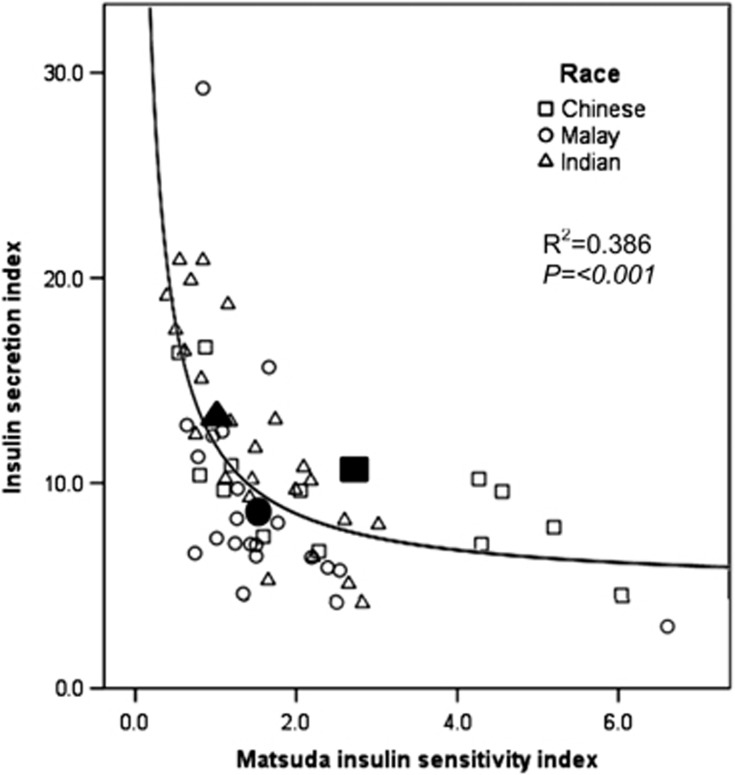
Relationship between insulin secretion index and ISI-Mat. Scatterplots of insulin sensitivity (measured by euglycemic-hyperinsulinemic clamp) vs insulin secretion index among Chinese (□), Malays (○) and Asian Indians (Δ). Filled symbols represent the mean values for each ethnic group. The non-linear regression line is shown for the hyperbolic fit of the data.

The oral DI was significantly higher in Chinese compared with Malays or Asian Indians (Figure 2). Figure 3 shows the hyperbolic relationship between beta-cell function (insulin secretion index) and insulin sensitivity (ISI-clamp). When we plotted the means of insulin secretion index and ISI-clamp by ethnicity, they were located at different points along the hyperbolic curve; Chinese and Asian Indians along the hyperbolic DI curve indicating appropriate beta-cell compensation for the prevailing insulin sensitivity, but Malays ‘falling off the DI curve', indicating a lower beta-cell response relative to insulin sensitivity.

## Discussion

In this study, we examined insulin sensitivity and beta-cell function between lean individuals of Chinese, Malay and Asian-Indian ethnicity. The findings from this study add to the body of evidence that in addition to differences in insulin sensitivity, postprandial glucose response and beta-cell function differ between Asian ethnic groups.

Our study showed that, among lean Asians, Malays exhibited higher postprandial glucose response but lower postprandial insulin response when compared with Asian Indians or Chinese. The insulinogenic index, a marker for early-phase insulin secretion, trended highest amongst Chinese, followed by Asian Indians and was the lowest amongst Malays. It has been suggested that a robust insulin response at the early phase after a meal is crucial in promoting hepatic glycogen storage and for the suppression of hepatic glucose production.^23^ A less robust insulin secretory capacity among the Malays might explain why this ethnic group displayed higher postprandial glucose excursion compared with Chinese or Asian Indians.

The euglycemic-hyperinsulinemic clamp directly measures whole-body glucose disposal and is regarded the gold standard against which all other measures of insulin sensitivity must be compared with. Surrogate markers of insulin sensitivity such as HOMA-IR reflects more of hepatic insulin sensitivity since fasting glucose is determined by hepatic glucose production, which itself is primarily regulated by insulin. The ISI-Mat is an insulin sensitivity index derived from a dynamic physiologic meal challenge in order to understand glucose-insulin responses.^21^ Our findings from ISI-clamp, HOMA-IR and ISI-Mat supported previous results by showing that Asian Indians were the least insulin sensitive, whereas Chinese were the most insulin sensitive.^6, 24^ In addition, Asian Indians had higher total adiposity (in particular abdominal adiposity) compared with the other two Asian ethnic groups. When we adjusted for waist circumference, the difference in insulin sensitivity between Chinese and Asian Indians remained significant (data not shown). An earlier study reported that among Japanese American men who developed T2DM, insulin insensitivity, increased insulin secretion and increased intra-abdominal fat were already present before the onset of glucose intolerance.^25^ This may help to explain why Asian Indians are more prone to developing T2DM and other cardio-metabolic diseases at a lower BMI and at younger age.^26^

Several studies reported that decreased insulin sensitivity and defective insulin secretion precede the onset of dysglycemia.^27, 28^ As long as the insulin sensitivity is matched by insulin secretion, normoglycemia is preserved, and a mismatch between these two parameters will therefore results in dysglycemia. This close relationship between insulin sensitivity and insulin secretion follows a hyperbolic curve, and the multiplication product of these two parameters is known as the DI.^9^ The DI therefore reflects beta-cell function relative to the prevailing insulin sensitivity,^29^ and predicts incident diabetes beyond fasting and 2-h glucose levels.^19, 20^ In prospective epidemiology studies, individuals who progressed from normoglycemia to T2DM exhibited the ‘falling off the DI curve' phenomenon, indicating a failure of insulin secretion to compensate for the degree of insulin resistance.^20^

In this study, we showed that the Chinese had the highest oral DI compared with Malays or Asian Indians. Malays exhibited lower beta-cell function (falling off the curve) compared with Chinese despite having similar insulin sensitivity. This suggests that Malays might have inadequate beta-cell secretory ability to compensate for their prevailing insulin sensitivity. We have further ascertained whether family history of diabetes might explain the lower beta-cell function amongst the Malays. The prevalence of first-degree relatives with diabetes mellitus in our study participants was 28.6% among Chinese, 19.0% among Malays and 16.7% among Asian Indians (data not shown), indicating that family history of diabetes does not explain why Malays have lower beta-cell function compared with Chinese. Several candidate gene regions for T2DM have been discovered by genome-wide association studies and confirmed in various populations worldwide.^30, 31^ These candidate gene regions are likely to influence beta-cell function. One of them is KCNQ1 gene polymorphism, in particular a risk allele r2283228 has been associated with a 1.7 times higher odds of developing diabetes mellitus among Malaysia Malays.^32^ Whether genetic polymorphism in the KCNQ1 gene or other diabetes candidate genes may account for the ethnic differences in beta-cell function warrants further investigations.

To our knowledge, there was no prior publication that compared beta-cell function in relation with insulin sensitivity between Malay ethnicity and other ethnic groups. The reason why Malays have lower beta-cell function compared with Chinese despite similar insulin sensitivity is not clear. The finding of low beta-cell function among Malays has important implications. Based on the National Health Survey in Singapore, the age-standardized prevalence rate of obesity is the highest among Malays and has increased from 19.1% in 2004 to 24.0% in 2010.^2^ Countries like Indonesia and Malaysia, where the majority population consists of Malay origin, have seen a rapid increase in the prevalence of obesity.^33, 34^ It is well established that insulin resistance is higher with greater obesity. Thus, with a background of compromised beta-cell function, it is probable that this select (Malay) population will see a greater rise in the prevalence of impaired glucose tolerance and diabetes mellitus with increasing obesity. A recent population forecast using an individual-level simulation model, based on Markov chain Monte Carlo methods demonstrated that the rising prevalence of obesity will double the prevalence of diabetes mellitus among Singaporeans by 2050, with Malays and Asian Indians being the most affected.^35^

Our study has several strengths. We studied three major ethnic groups (Chinese, Malays and Asian Indians) that represented the majority of ethnic groups living in Asia, a region where the prevalence of T2DM and cardiovascular disease are expected to increase over the next several decades.^36^ Our subjects were young, healthy and lean males, which allow us to determine the metabolic responses following a liquid mixed-meal challenge prior to the onset of chronic diseases. We have used the gold standard for measurement of insulin sensitivity using the euglycemic-hyperinsulinemic clamp technique.^37, 38, 39^ We also carried out the liquid mixed-meal challenge to investigate insulin secretory capacity instead of using an oral glucose tolerance test. As a mixed-nutrient load, the liquid mixed meal provides a more physiologic stimulus for assessing glucose and insulin homeostasis.^15, 16, 17, 18^ Furthermore, the liquid mixed meal does not require chewing, thus it will not be a potential confounding factor in the post-meal glucose or insulin response.^40^

There are also limitations in this study. We recognize that the number of subjects in this study was small, however they were well-matched for age, BMI and other metabolic parameters. The insulin sensitivity, as determined by hyperinsulinemic-euglycemic clamp study, agrees with previous publications that showed a statistical difference between Chinese and Asian Indians. Nonetheless, future study with a larger sample size and inclusion of lean females is needed to validate these results. Differences in the nutrient absorption might also contribute to the ethnic differences in the post-meal glucose response. All of our subjects did not have any history of malabsorption nor was there evidence of lactose intolerance, thus we believe that differences in nutrient absorption play a minimal role in our study findings. Future studies should look into differences in nutrient absorption and the role of stool metagenomics in mediating the ethnic differences in postprandial glucose and insulin response. We did not measure incretin responses, which might contribute to the differences in the post-meal insulin response. However, one study showed that the higher early insulin response in African Americans compared with European Americans was not due to differences in circulating incretin concentrations.^41^ Interpretation of insulin secretion and insulin sensitivity must also take into account hepatic insulin extraction and hepatic insulin sensitivity, both of which were not examined in this study.

In summary, we have shown that, in addition to ethnic differences in insulin sensitivity, lean Chinese, Malays and Asian Indians also exhibit differences in beta-cell function. While lean Chinese and Asian Indians show appropriate beta-cell function in relation to insulin sensitivity, lean Malays exhibit lower beta-cell function relative to their prevailing insulin sensitivity. With this background, Malays may face a rapid increase in the incidence of diabetes with rising prevalence of obesity, and that measures to maintain healthy body weight would be a key strategy to mitigate the development of T2DM in this population.

## Figures and Tables

**Table 1:** Baseline characteristics of study subjects by ethnicity

	*Chinese (*n=*14)*	*Malay (*n=*21)*	*Asian Indians (*n=*24)*	P*-value (age adjusted)*
Age (years)	29.6 (1.7)^a,b^	25.2 (0.7)^a^	24.3 (0.7)^b^	—
BMI (kg m^−^^2^)	21.5 (0.4)	20.9 (0.3)	21.6 (0.3)	0.212
Waist circumference (cm)	75.8 (1.1)^a^	73.9 (1.0)^b^	78.7 (0.9)^a,b^	<0.001
Percent body fat	19.4 (1.4)	17.5 (1.0)^a^	20.4 (1.0)^a^	0.024
Fasting glucose (mmol l^−1^)	4.41 (0.09)	4.12 (0.10)	4.12 (0.07)	0.382
Fasting insulin (mU l^−1^)	4.85 (0.65)	5.93 (0.90)	7.54 (0.92)	0.103
Total cholesterol (mmol l^−1^)	5.10 (0.27)	4.60 (0.17)	4.43 (0.16)	0.513
LDL-cholesterol (mmol l^−1^)	3.27 (0.24)	2.86 (0.15)	2.76 (0.12)	0.650
HDL-cholesterol (mmol l^−1^)	1.34 (0.07)	1.34 (0.04)	1.33 (0.05)	0.974
Triglycerides (mmol l^−1^)	1.08 (0.13)	0.86 (0.06)	0.76 (0.07)	0.284
Aspartate aminotransferase (U l^−1^)	20.1 (1.5)	22.1 (1.3)	22.5 (1.0)	0.158
Alanine aminotransferase (U l^−1^)	17.9 (2.1)	18.7 (1.4)	19.0 (1.1)	0.190

Abbreviation: BMI, body mass index.

Data are presented as mean (SE). *P*-value for comparison among the ethnic groups adjusted for age.

Mean values within each row with same superscript letters were significantly different (*P*<0.05).

**Table 2:** Measures of insulin sensitivity and pancreatic beta-cell function

	*Chinese (*n=*49)*	*Malay (*n= *31)*	*Asian Indians (*n= *29)*	P*-value (age adjusted)*
HOMA-IR	1.31 (0.08)^a^	1.40 (0.19)	2.00 (0.31)^a^	0.003
HOMA-B	133.0 (8.9)^a^	156.2 (21.0)^b^	284.6 (53.2)^a,b^	0.008
				
*Euglycemic clamps derivative*				
Insulin sensitivity index (ISI-clamp) per kg lean mass (mg kg^−1^ min^−1^ mU^−1^ l^−1^)	14.2 (1.2)^a^	12.9 (1.2)^b^	8.8 (0.6)^a,b^	0.001
				
*LMMTT derivatives*				
Incremental area under curve—glucose (mmol•min l^−1^)	157.0 (31.5)^a^	306.0 (29.5)^a^	224.6 (23.4)	0.002
Incremental area under curve—insulin (mU•min l^−1^)	11138 (1272)	9963 (1316)	13061 (1282)	0.188
Insulinogenic index	83.3 (41.7)	34.6 (7.1)	51.9 (5.6)	0.057
Insulin secretion index (mU mmol^−1^)	10.5 (1.2)	9.1 (1.2)	12.3 (1.1)	0.136
ISI-Mat	2.70 (0.49)^a,b^	1.69 (0.28)^a^	1.51 (0.16)^b^	0.029

Abbreviations: HOMA-B, homeostatic model assessment—beta-cell function; HOMA-IR, homeostatic model assessment—insulin resistance; ISI-Mat, matsuda insulin sensitivity index; LMMTT, liquid mixed-meal tolerance test. Data are presented as mean (SE). *P*-value for comparison among the ethnic groups was adjusted for age or waist circumference. Mean values within each row with same superscript letters were significantly different (*P*<0.05).
