# Supplementary material for: Ethnic differences in insulin sensitivity and beta-cell function among Asian men
Source: Nutr Diabetes. 2015 Jul 20;5(7):e173–. doi: 10.1038/nutd.2015.24 (PMC4521178; doi:10.1038/nutd.2015.24)
Supplement: Supplementary Figure 1 [file nutd201524x1.doc]

**Supplementary Figure 1 -** Incremental change in plasma insulin concentration response following the liquid mixed meal tolerance test by ethnicity.


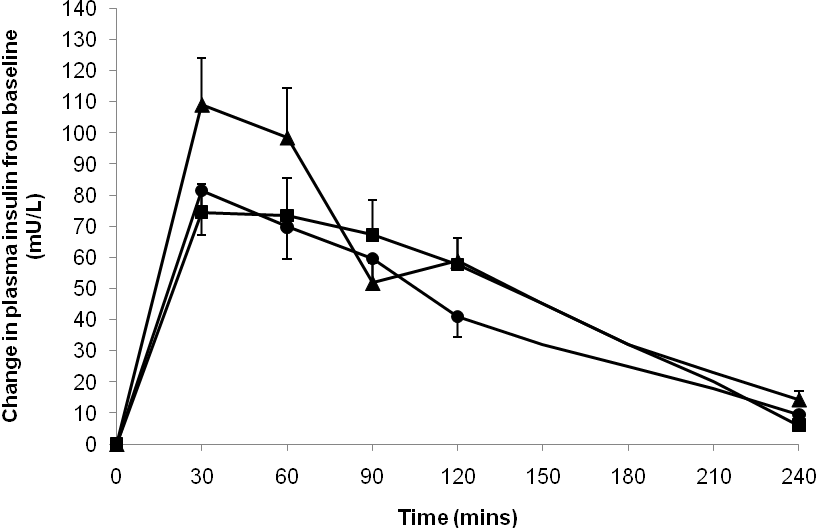


Incremental change in plasma insulin concentration response following liquid mixed meal tolerance test Chinese ( ), Malay ( ) and Asian-Indians ( ). P interaction for ethnic groups x plasma insulin glucose response = 0.164 (adjusted for age).
